# COVID-19, Chikungunya, Dengue and Zika Diseases: An Analytical Platform Based on MALDI-TOF MS, IR Spectroscopy and RT-qPCR for Accurate Diagnosis and Accelerate Epidemics Control

**DOI:** 10.3390/microorganisms9040708

**Published:** 2021-03-30

**Authors:** Jéssica Costa, Eugénio C. Ferreira, Cledir Santos

**Affiliations:** 1 Programa de Doctorado en Ciencias de Recursos Naturales, Universidad de La Frontera, Temuco 4811-230, Chile; j.souza01@ufromail.cl; 2 CEB-Centre of Biological Engineering, Universidade do Minho, Campus of Gualtar, 4710-057 Braga, Portugal; ecferreira@deb.uminho.pt; 3 Department of Chemical Science and Natural Resources, Universidad de La Frontera, Temuco 4811-230, Chile

**Keywords:** diagnostic methods, emerging diseases, viral infection

## Abstract

COVID-19 and arboviruses (ARBOD) epidemics co-occurrence is a great concern. In tropical and subtropical regions, ARBOD diseases such as chikungunya, dengue, and Zika are frequent. In both COVID-19 and ARBOD cases, an accurate diagnosis of infected patients is crucial to promote adequate treatment and isolation measures in COVID-19 cases. Overlap of clinical symptoms and laboratory parameters between COVID-19 and ARBOD present themselves as an extra challenge during diagnosis. COVID-19 diagnosis is mainly performed by quantitative reverse polymerase chain reaction (RT-qPCR), while ARBOD diagnosis is performed by serology, detection of antigen or antibody, and molecular diagnosis. In this review, the epidemiologic profile of arboviruses and SARS-CoV-2 is analyzed, and potential risks of symptom overlap is addressed. The implementation of an analytical platform based on infrared (IR) spectroscopy, MALDI-TOF mass spectrometry, and RT-qPCR is discussed as an efficient strategy for a fast, robust, reliable, and cost-effective diagnosis system even during the co-occurrence of virus outbreaks. The spectral data of IR spectroscopy and MALDI-TOF MS obtained from COVID-19 infected and recovered patients can be used to build up an integrated spectral database. This approach can enable us to determine quickly the groups that have been exposed and have recovered from COVID-19 or ARBOD, avoiding misdiagnoses.

## 1. Introduction

Emerging infectious disease (EID) is conceptualized as an abrupt rise of new pathogen in a host population [1]. Further, the term also extends to re-emergent pathogens that have a sharp incidence in a new geographical area [1]. Overall, EID encompasses a diversity of pathogenic microorganisms (bacteria, fungi, viruses, and so forth) that may have an animal origin, so-called zoonosis, and also be linked to other sources (e.g., food-borne and water-borne pathogens) [2]. However, the increase of EIDs has spilled over from animals to humans in the last decade and has received attention from health agencies worldwide.

Emerging zoonosis corresponds to transmissible infections from vertebrate animals to humans [3]. According to Jones et al. [2], around 60% of EIDs are originated from animals, which include domesticated species, free-range wildlife, and wild animals reared by man [4]. Recent studies have suggested that zoonotic spillover is related to human footprint [5,6,7]. The anthropogenic land conversion, industrial growth, and climate change events can either result in, or be an open avenue to, deforestation, habitat fragmentation, and invasion of ecological wildlife niches by intensified farming and animal husbandry [5,6,7].

All these shifts in landscape disrupt ecological balance, affecting species distribution and also food supply [8]. Hence, these can drive zoonotic sources (e.g., bats, ungulates, rodents, and so forth) into new areas, promoting virus spread by contact with potential intermediate hosts (e.g., bat, rabbit, cattle, pigs, livestock, and so forth) or disease vectors (e.g., *Aedes* mosquitos) [8]. Finally, viral pathogens can jump to humans.

The coronavirus disease 2019 (COVID-19) outbreak caused by the severe acute respiratory syndrome coronavirus 2 (SARS-CoV-2) became a pandemic with fast spread worldwide [9]. The origin of SARS-CoV-2 has been closely related to zoonotic sources. *Rhinolophus* bats are a major candidate [10], although till now, animal reservoirs and the turning point of spillover event have as yet been unknown [4,11].

The COVID-19 pandemic has caused an unprecedented world economic crisis. Severe, intermediate, and mild COVID-19 symptoms have been manifested in different groups, overloading healthcare facilities [9]. Throughout 2020, the global economic decline was 4.3%, although this contraction was perceived more harshly in developing economies [12]. Sectors of service and consumption related to tourism are among the most affected, having a direct impact on employability. According to the International Labour Organization [13], about 255 million full-time jobs have been lost. Concomitant COVID-19 occurrence with other viral epidemics have been causing concern, mainly in low-income countries [12].

Arbovirus diseases (ARBOD) are caused by arboviruses mainly maintained in nature, or to an important extent, through biological transmission between susceptible vertebrate hosts by hematophagous arthropods [14]. Simultaneously, the co-circulation risk of arthropod-borne virus (arbovirus), causal agents of ARBOD such as chikungunya, dengue, and Zika diseases, imposes an extra burden on health systems. Although this panorama can normally be observed mainly in tropical regions’ endemics for some ARBOD [15], France and Italy have recently reported autochthonous dengue infection cases in the year 2020 [16].

The co-occurrence of different emerging virus-based diseases is a challenge from an epidemiological point of view. The similarity of symptoms, cases of virus co-infection, and cross-reaction can result in a misdiagnosis [17,18].

Thus, in a pandemic setting, rapid and accurate approaches are necessary to speed up time for diagnosis and for keeping the results reliable. As a standard technique, the quantitative reverse polymerase chain reaction (RT-qPCR) has been widely used in viral disease diagnosis. Alternatively, IR spectroscopy and MALDI-TOF MS have been introduced as routine tools in microbiology laboratories, being used for identification of bacteria, fungi, and viruses. The usability of these techniques can cover identification of transmitting vector, pathogen agent, and also the virus detection in final receptor [19,20,21].

In this review, the epidemiologic profile of arboviruses (chikungunya, dengue, and Zika) and SARS-CoV-2 is analyzed, and the potential risks of symptom overlap is addressed. Further, the implementation of an analytical platform based on IR spectroscopy, MALDI-TOF mass spectrometry, and RT-qPCR is discussed as an efficient strategy for a rapid and accurate diagnosis even during the co-occurrence of virus outbreaks.

## 2. Arbovirus Disease

Zika, chikungunya, and dengue are tropical and subtropical diseases caused by arboviruses that pose a major concern for global public health [22]. The interplay between pathogens (one or more virus serotypes), transmission vectors (*Aedes* mosquitoes), maintenance host, and humans are pivotal to the success of arboviruses cycle infection [23].

First, the enzootic cycles are restricted to the interplay among arbovirus-transmitting mosquitoes and maintenance hosts such as nonhuman primates (e.g., baboons, green monkeys) and other small mammals (e.g., rodents, bats) [23]. The following step is the infection overflows for humans, which initially occur in forests, and then are amplified into urban areas [24].

The first reported dengue outbreaks were in Asia, while for chikungunya and Zika, it was Africa [25,26,27]. Then, a rapid spread occurred in intra-continental cycles of disease re-emergence. Afterward, over different periods, a quick global spread was observed [28,29,30]. According to the WHO [31], dengue is endemic in more than 128 countries. The number of dengue cases increased over 8-fold over the last two decades, from 505,430 to 4.2 million [31]. Furthermore, it is estimated that approximately 390 million cases of symptomatic dengue infections annually lead to about 10,000 deaths per year [31].

The American continent is a hotspot for dengue, with ongoing resurface in annual peaks [32]. A recent upsurge of chikungunya virus (CHIKV) cases has been reported in Africa (Democratic Republic of Congo, 2019), Europe (Italy, 2017), and America (Brazil) [33].

In 2014, Brazil was an epicenter of infections by CHIKV and Zika virus (ZIKV) cases overflow, with several points of arbovirus co-emergencies [34,35]. A key factor for the spread of these diseases lies in the ability of the *Aedes* mosquito main propagation vectors to reproduce and adapt quickly to peridomestic ecotope [36]. Throughout this urban cycle, humans act as virus reservoirs [24].

All these arboviruses are under constant surveillance by regulatory agencies worldwide. The WHO set chikungunya and dengue on the current list of neglected tropical diseases (NTDs) [37]. In the European Union (EU), the European Centre for Disease Prevention and Control (ECDC) monitors arthropod vector distribution and also cases of human transmission. Dengue virus (DENV) has been in tighter control than ZIKV and CHIKV. The last two are rare in EU, and the noted cases are mainly traveler-associated [38].

In the United States, the National Institute of Allergy and Infectious Diseases (NIAID’s) and the Centers for Disease Control and Prevention (CDC) insert these arboviruses on the pathogen priority list [39,40]. In South America, this monitoring has been carried out by the combined task force of government agencies and the Pan-American Health Organization [41].

### 2.1. Arbovirus

DENV and ZIKV are both positive-sense, single-stranded RNA viruses belonging to the *Flaviviridae* family [42]. DENV exists as four serotypes (DENV1=4) and infection with any serotype may be asymptomatic or can result in mild to severe clinical symptom [29]. CHIKV belongs to the family *Togaviridae*, genus *Alphavirus*. It is a positive-sense, non-segmented, single-stranded RNA (12 kb in length) virus, with an enveloped icosahedral capsid [43]. Three main lineages of CHIKV have been identified and comprise the enzootics East/Central/South African (ECSA), West African, and Asian strains genotype [44]. In the American continent, the chikungunya epidemic was caused mainly by the CHIKV-Asian genotype. However, the ECSA-genotype was detected in northeast Brazil [45].

### 2.2. Arbovirus Disease Transmission

The primary transmission mechanism of CHIKV, DENV, and ZIKV is through *Aedes aegypti* and *Aedes albopictus*, although non-vector transmission has been reported. Rarely cases have reported CHIKV and DENV infection vertically during pregnancy and via blood-borne transmission [46,47,48]. Several cases of ZIKV-infected pregnant women have resulted in congenital and postnatal modification due to intrauterine infection [49].

Between 2015 to 2017, Brazilian Ministry of Health notified 2639 cases of microcephaly by Zika disease, pointing out possible underreporting for other newborn congenital malformations [49]. Conversely, few case reports have linked CHIKV and DENV to vertical transmission during pregnancy [46,47,48].

Isolating arbovirus from body fluid (e.g., urine, saliva, and breast milk) of infected individuals is a feasible possibility, even though, until now, no transmission from these sources have been notified [50].

### 2.3. Arbovirus Disease Symptoms

**Figure 1:**
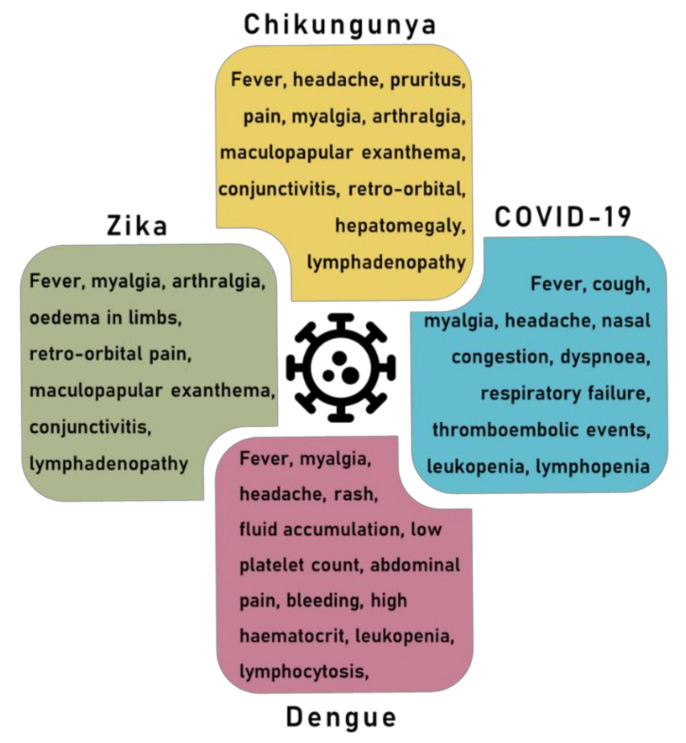
The main symptoms related to COVID-19, dengue, Zika, and chikungunya.

The main clinical symptoms of CHIKV, DENV and ZIKV are listed in Figure 1. Symptoms typically appear after an incubation time of 4–7 days. Overall, DENV has been reported as more lethal than ZIKV and CHIKV [51], even though the latter two, in the long term, may show the gravest disease progression [52,53].

In more severe manifestations, DENV can cause dengue hemorrhagic fever and dengue shock syndrome [29]. ZIKV is linked to congenital malformations, encephalitis, and Guillain–Barré syndrome (GBS) [53,54], whereas debilitating polyarthralgia is recurrent in 30–40% of CHIKV-infected individuals [55].

For these arboviruses, clinical manifestations such as fever, exanthema, conjunctivitis, retro-orbital headache, and arthralgia are similar, mainly during the acute phase [56]. Symptoms overlapping mainly during arbovirus co-circulation are a critic outlook.

A triple epidemic scenario has already been faced in arbovirus hotspots regions of Brazil, leading to misdetection and disease frequency misreporting [15,57,58]. It is important to note that sudden appearance of CHIKV, DENV, and ZIKV, as well as other seasonal respiratory tract diseases (e.g., H1N1, rhinoviruses, respiratory syncytial virus), and also the current pandemic with SARS-CoV-2, will continue to occur.

The co-occurrence outbreaks remain a great pressure on the public health systems, which can get overloaded [15,59]. This scenario poses a further challenge for health systems, that should be able to accurately diagnose and treat single and co-infection cases [59].

### 2.4. Vaccine Development against Arbovirus

Up to now, there is no licensed vaccine available against CHIKV and ZIKV. However, for ZIKV, several candidate vaccines are in ongoing trials (phase I and II) [60,61]. For DENV, a current vaccine in pre-clinical phase adopts different development paths such as a live-attenuated virus, inactivated virus, recombinant protein, DNA vaccine, viral vector vaccine, and heterologous prime/boost vaccines [62]. Only the vaccine CYD-TDV (Dengvaxia^®^, Sanofi Pasteur, Lyon, France) has been licensed in the countries of Asia, Latin America, and the Pacific [63].

International drug regulatory agencies such as the European Medicines Agency (EMA) and the Food and Drug Administration (FDA) have also approved the use of CYD-TDV in DENV-seropositive individuals [64]. The CYD-TDV vaccine performance depended on prior sero-status, the efficacy among DENV seropositive individuals ranging from 42.3% to 77.7% depending on DENV serotype [63,65]. In seronegative participants, CYD-TDV is less effective and increases the risk of severe dengue symptoms in an eventual subsequent infection [63,64,66].

Alternatively, there are second-generation dengue vaccines (TAK-003, TDV, Takeda); TV003/TV005, National Institutes of Health, United States) that, in phase 1 and 2, proved to be well-tolerated and immunogenic against all serotypes. Phase 3 efficacy trials are currently ongoing [65].

## 3. Coronavirus Disease

Coronavirus disease 2019 (COVID-19), caused by the severe acute respiratory syndrome coronavirus 2 (SARS-CoV-2), became one of the major outbreaks of the century [9]. SARS-CoV-2 emerged in late 2019 and spread to more than 220 countries within a short period, resulting currently in more than 2,564,560 deaths and more than 115,289,961 confirmed cases [67].

Previous coronavirus outbreaks such as (SARS)-CoV and the Middle East respiratory syndrome (MERS)-CoV have occurred from time to time. Due to severe symptoms, both of them are considered public health concerns [68]. Other low-pathogenicity human coronaviruses (HCoVs) such as HCoV-29E, HCoV-HKU1, HCoV-OC43, and HCoV-NL63 have been already identified as responsible for upper and minor respiratory tract infections [69].

SARS-CoV-2 is a positive-sense single-stranded enveloped RNA virus. It is clustered in the genus *Betacoronavirus*, sharing 79% genome sequence identity with SARS-CoV and 50% with MERS-CoV [70]. Although genetically similar, SARS-CoV-2 has a profile of clinical signs and transmission efficiency distinct from SARS-CoV-like coronaviruses [71].

Despite some uncertainties about the transmission starting point, bats are reported as natural reservoirs of SARS-CoV-2 [10]. Initially, pangolins were suggested as intermediate hosts, but this hypothesis was discharged [72,73]. As a zoonotic disease, SARS-CoV-2 can be transmitted from animal to animal, animal to human, and also human to human [74]. The intermediate species that promoted the spread of these disease to human is not yet known [74].

Animal-to-human transmission was punctual, being linked to the seafood market in Wuhan, where wild animals were also sold [74]. However, the massive spread of the illness is by person-to-person contact through small droplets produced when people cough, sneeze or talk [75]. Cases of intrauterine transmission have been reported [76,77]. SARS-CoV-2 has been detected in breast milk [78], stool [79], blood, and urine samples [80], but transmission through these routes remains unclear.

Nosocomial setting has been reported as an important source of infection. According to Houlihan et al. [81], 84 out 200 (44%) frontline healthcare workers from a London hospital (United Kingdom) were infected with SARS-CoV-2. Transmission through contaminated surfaces has been suggested, since SARS-CoV-2 has been detected for up to 7 days on surfaces (e.g., plastic, stainless steel, copper, and cardboard), but till now the data regarding indirect virus transmission are inconclusive [82].

After infection, generally, SARS-CoV-2 viral loads reach a peak within the first week after symptom onset which entails that transmission highest risk occurs in the very early disease stage [83,84]. Thus, immediate isolation measures during the first symptoms are essential, since the high titers of SARS-CoV-2 at the onset of disease possibly increase the virus transmission efficiency [83].

SARS-CoV-2 has shown greater transmissibility than other viruses’ diseases, such as MERS-CoV and SARS-CoV-1 [71]. Several studies sought to estimate the basic reproduction number (R_0_) in different populations. R_0_ corresponds to an average number of secondary infections arising from a primary infected person [85].

WHO [86] have estimated R_0_ values for SARS-CoV-2 ranging between 1.4–2.5. However, several studies reached different values ranged from 1.94 to 6.94. R_0_ above 1 indicates that the transmissibility process will continue to happen. R_0_ is an important parameter to drive the appropriate control measures [87].

COVID-19 clinical manifestation ranges from asymptomatic to severe cases. The most common symptoms at illness onset are fever, fatigue, and dry cough, while nasal congestion, rhinorrhea, sore throat, and myalgia are reported less often [88,89].

In severe cases, respiratory symptoms such as breath shortness and pneumonia make up the framework of acute respiratory distress syndrome (ARDS), the main mortality cause [90]. Non-respiratory symptoms such as palpitation, diarrhea, and headache have been reported. SARS-CoV-2 infection might also have neurotropic potential, until now 16 cases of Guillain–Barré syndrome (GBS) in a para-infectious (3) and post-infectious (13) profile have been associated with SARS-CoV-2 [91]. However, it is necessary to expand the epidemiological data to support a causal relationship [92].

SARS-CoV-2 mutation rates, as well the evolutionary convergence of different strains from different locations, have raised a red flag for the scientific community [93]. Although, until now, it is suggested that vaccines are equally effective for all SARS-CoV-2 strains [93].

The new variant of SARS-CoV-2 in the UK is estimated to be up to 70% more transmissible than the previous one [94,95]. Other variants detected in South Africa (e.g., 501Y.v2 or B1351) and Brazil (B.1.1.28.1) underscore that there is much room for improvement in the understanding of the pathogenicity and action mode of SARS-CoV-2.

Several technologies have been used in COVID-19 vaccine development. Currently, two RNA-based vaccines (tozinameran from Pfizer–BioNTech, Marburg, Germany and mRNA-1273 from Moderna, Massachusetts, United States), two conventional inactivated virus vaccines (BBIBP-CorV from Sinopharm, Beijing, China and CoronaVac from Sinovac, Beijing, China), and one viral vector vaccines (Sputnik V from the Gamaleya Research Institute, Moscow, Russia) already have had the first doses applied [93].

## 4. Overlapping Symptoms and Co-Infection

The current pandemic has unleashed extra pressure on public health systems, making itself even more threatening in regions that are endemic for arboviruses. Simultaneous to SARS-CoV-2, arbovirus infections continue spreading, mainly in tropical settings such as Southeast Asia and Latin America [29].

Currently, Brazil is the second country with the highest number of deaths due to COVID-19 [96]. Manaus, the capital of Amazonas State in northern Brazil, is currently classified as a purple area (Tier 1), which characterizes an extremely critical situation. Concomitantly, dengue reaches its peak in the first quarter of the year in Manaus and other municipalities in northern Brazil that are currently also classified as purple and red for COVID-19 [97]. Other geographical regions such as India, Thailand, and Singapore faced this overlap of COVID-19/ARBOD infections between September and November 2020 [98].

**Figure 2:**
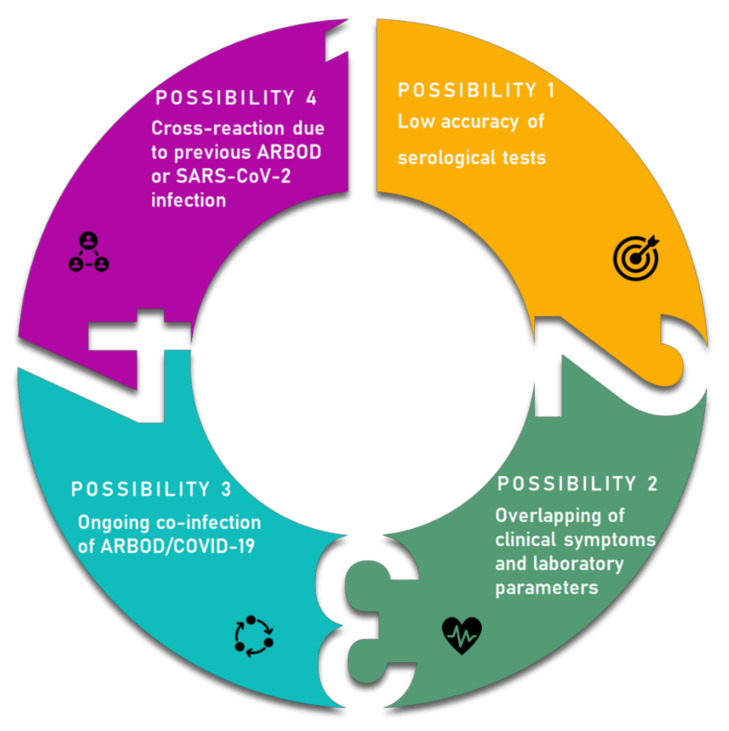
The main hypotheses that may be related to misdiagnosis between COVID-19 and arboviruses (ARBOD).

The overlap of COVID-19/ARBOD, besides overloading health centers, can result in misdiagnosis, as observed in Figure 2. During the disease onset, COVID-19 and dengue, for instance, shared similar clinical and laboratory features, being difficult to distinguish from each other. The initial clinical symptoms (e.g., fever, myalgia, fatigue, chills, and headache) and laboratory parameters (e.g., lymphopenia, leukopenia, thrombocytopenia, and elevated transaminases) can be similar in both illnesses [99].

Furthermore, diagnosis based only on physical features may be insufficient in some cases. Joob et al. [100] reported a case of a patient who presented a skin rash with petechiae and low platelet count, a common clinical finding in dengue illness. Later, the patient showed respiratory problems, so COVID-19 infection was confirmed by RT-PCR. Similarly, Yan et al. [101] reported two cases of patients with COVID-19 who firstly produced false-positive dengue results in a rapid serological test. Misdiagnosis of COVID-19 and dengue may delay the appropriate treatment, as well as the determination of patient isolation in COVID-19 cases. Besides, it can prompt the risk of transmission in a nosocomial setting.

Prasitsirikul et al. [102] reported a possible infection of a nurse by SARS-CoV-2 during patient blood sampling. The patient had mild thrombocytopenia and IgG and IgM positive for dengue, but the symptoms progressed to breath shortness and pulmonary reticular infiltration. Afterwards, RT-PCR was carried out confirming the positive result for SARS-CoV-2. These inconsistencies have been generating concern about the reliability of rapid diagnostic tests.

Another hypothesis is that cross-reactivity among DENV and SARS-CoV-2, which may be related to tandem virus infection, can lead to false-positive dengue serology [17,18,98,99,101,102,103,104,105]. Both hypotheses are feasible and require more comprehensive cohort studies.

Against this background, public health management agencies are responsible for tackling the current COVID-19 pandemic and also for predicting and preventing the concomitant risk of emerging ARBOD infections. It suggests that such agencies must primarily focus on setting up a nationwide platform for (a) identifying and detecting viral pathogen, and (b) monitoring viral load in both infected symptomatic and asymptomatic, and in recovered patients. An implementation of an analytical platform is an efficient strategy for accurate diagnosis, accelerating epidemics control [106,107].

## 5. Integrated Analytical Platform for Fast and Cost-Effective of EID Diagnosis

Enzyme-linked immunosorbent assay (ELISA) which detects specific antibodies from human serum has been widely used for fast detection of virus diseases. ELISA offers a rapid result with a good cost–benefit ratio, although in endemic areas of chikungunya, dengue, and Zika, cross-reactivity is expected in diagnoses [14,58]. Further, cases of false-negatives for COVID-19 have been reported, highlighting low accuracy and precision as important drawbacks [17,18,98,101]. A fast, simple, and low-cost analysis tool which must be effective in screening for virus variants is urgently required.

Until now, RT-qPCR testing was considered as the golden method for screening cases of COVID-19 and also chikungunya, dengue and Zika [21]. However, in a pandemic context, where a large number of analyses are required in a short time, these tests cannot be cost-effective. RT-qPCR per-test costs are approximately USD 10 and reaction times require about 2 h to perform [108,109]. This approach could be optimized if it worked conjugated with other techniques.

Infrared (IR) spectroscopy techniques can be a useful tool for the early diagnosis and monitoring of virus human infections [21]. Rapid diagnosis of COVID-19 by IR spectroscopy offers reduced dependence on RT-qPCR technique. IR spectroscopy can detect chemical bonds of structural components of microorganisms and can reach detection limit in the concentration range of 5–700 ppb [110,111].

**Figure 3:**
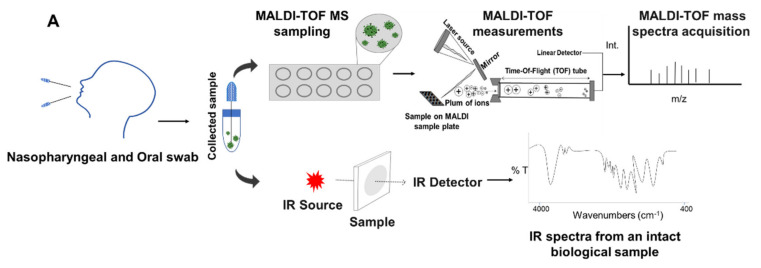

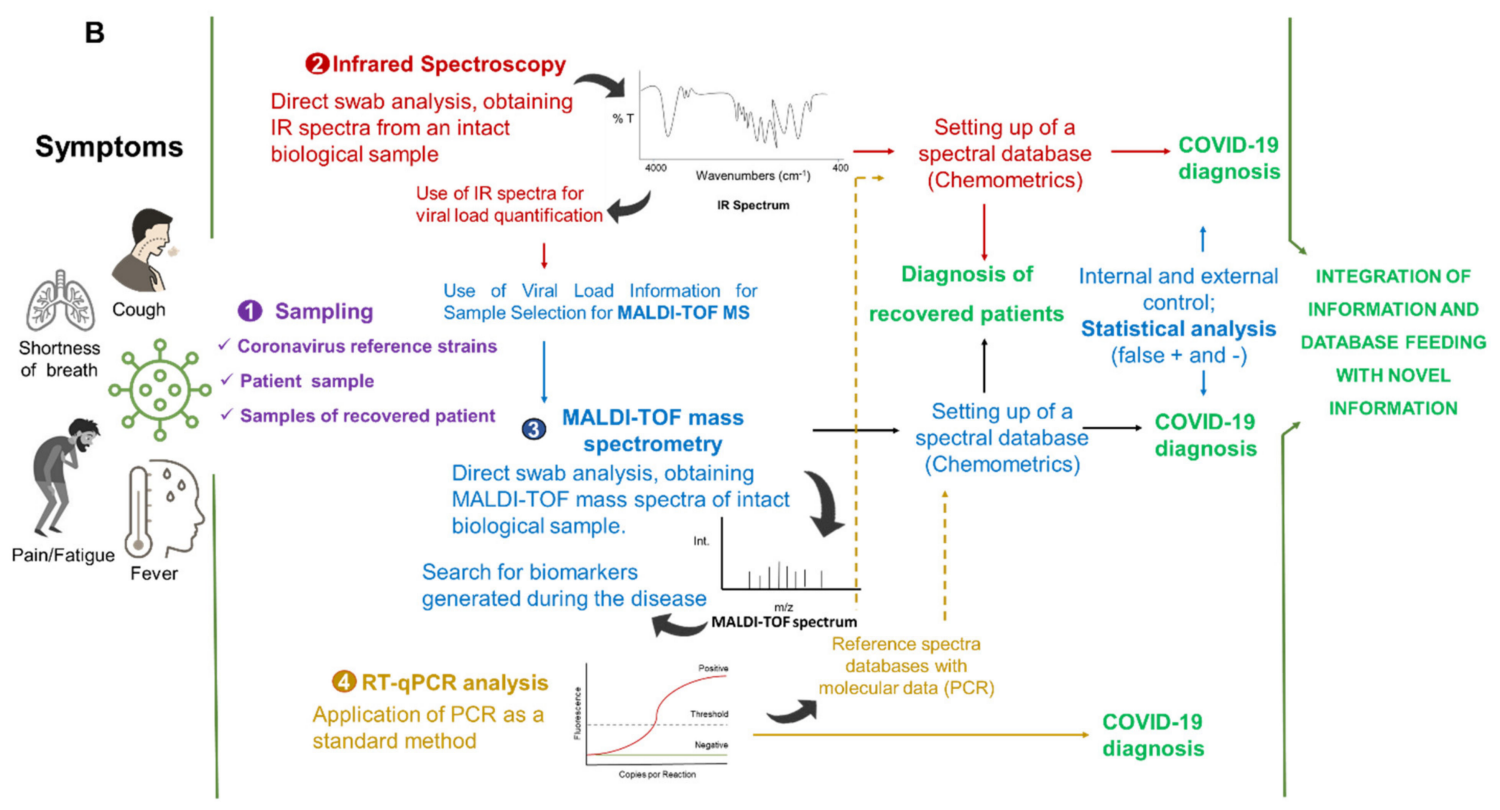
Integrated analytical platform based on IR spectroscopy, MALDI-TOF MS, and RT-qPCR for SARS-COV-2 detection and quantification and COVID-19 fast diagnosis. (**A**) General procedure for nasopharyngeal and oral swab sampling and spectral acquisition. (**B**) Way for integration of information and database feeding with novel information. RT-qPCR is the gold standard of the proposed analytical platform.

An IR spectral database for COVID-19 diagnosis must be built up based on RT-qPCR-validated IR spectra by using SARS-CoV-2 reference and clinical strains. Such an IR spectral database must then be fed with clinical strains as it has been made before for other kind of microorganisms, which includes viruses [111,112,113,114]. Once the database is created, COVID-19 detection based on IR spectroscopy does not require reagents for spectral acquisition, configuring a fast and inexpensive method [112] (Figure 3A).

IR spectroscopy can also be used to quantify SARS-CoV-2 viral load in carriers of the virus (Figure 3B). This data is important since the virus transmission capacity is directly related to its load. Furthermore, in most cases, viral load is strictly related to different disease cycles [84]. Once it does not require chemicals for analysis (basically manpower), applying the IR spectroscopy approach to control spread of SARS-CoV-2 is a simple and cost-effective procedure.

In addition to manpower, the cost for a single IR spectroscopy measurement is basically that related with swab and electricity [111]. Moreover, the results are delivered in up to one minute from the reading of the sample by the equipment. The rapid test can be applied for workers who enter and leave their work and for people who enter and exit areas of public places, such as public and private companies, factories, clinical offices, airports, and bus stations.

Similarly, matrix assisted laser desorption ionization–time-of-flight mass spectrometry (MALDI-TOF MS) is a highly sensitive technique efficient for SARS-CoV-2 detection by swab analysis [112] (Figure 3A). In addition, detection of SARS-HCoV-OC43 has also been achieved in collaborative testing of RT-qPCR and MALDI-TOF MS assays [115]. MALDI-TOF MS can detect traces of organic molecules at concentrations from femtomolar to attomolar level (10–15 to 10−18 mol/L) and is able to establish fingerprints of biomarkers as lipids and proteins, expressed in an infectious process assay [116].

Nachtigall et al. [112] used the direct swab procedure to obtain MALDI-TOF mass spectra from a total of 362 samples. Samples were previously analyzed by RT-qPCR and confirmed as SARS-CoV-2-positive (211 samples) and negative (151 samples). According to the authors, detection of SARS-CoV-2 in nasal swabs using MALDI-TOF MS was succeeded with an accuracy of about 94% in COVID-19 diagnosis.

Mass spectrometry-based approach to ARBOD diagnosis is ongoing, although until now few studies have targeted this tool to ZIKV, CHIKV, and DENV infection analysis [117,118]. For these arboviruses, previous results highlighted that there are specific marker ions which can be used to define rapid diagnosis by mass spectrometry [118]. Likewise, in cases of patients (*n* = 3) co-infected, it was possible to establish the metabolic fingerprint for CHIKV, DENV II, and ZIKV [118].

Although MALDI-TOF is a high-performance mass spectrometry tool, it is still underutilized in arbovirus detection. Conversely, MALDI-TOF MS has widely been applied in diagnosis of respiratory (influenza viruses), enterohepatic (hepatitis virus), and herpesvirus infections [107,119]. Further, MALDI-TOF MS conjugated with molecular (PCR) approach has been achieving high-throughput virus detection.

Cost of reagents and consumables per sample for bacterial infection diagnosis by MALDI-TOF MS is estimated to be around USD 0.43 [120]. Similarly, regarding SARS-CoV-2 analysis and COVID-19 diagnosis, MALDI-TOF MS reagents are cheaper than those used in RT-qPCR tests [112].

Regarding time of diagnosis for influenza virus, for IR spectroscopy it has been achieved by 1 min/per sample [111]; while for MALDI-TOF MS it has been achieved for 3 h/per sample [121]. Unlike the aforementioned techniques, RT-qPCR is costly and often time-consuming. If using a reliable spectra database, IR spectroscopy and MALDI-TOF MS techniques are fast and reliable methods for SARS-CoV-2 analysis and COVID-19 diagnosis.

In addition, IR spectroscopy appears as a reliable method for the virus load quantification. Both techniques are complementary to each other, even in the case that the equipment are based in different laboratories or physical spaces. Molecular biology-based RT-qPCR must be used as the gold standard for such analytical platform, and the database must be integrated using a common chemometrics language (Figure 3B).

Analysis based on IR spectroscopy has presented the highest accuracy, close to 97% (29 of 30 samples) and at 100% (30 of 30 samples), for non-influenza and influenza patients, respectively, in both cases, using direct test based on nose swab analysis [111].

The best COVID-19 surveillance approach is by testing and isolating new cases and tracing their contacts. Using this approach, a huge resurgence of infections can be avoided [122]. It is necessary to be prepared with an integrated analytical platform that operates with low-cost analysis and high confidence level, able to support clinical decision-making.

As can be observed in Figure 3A,B, an integrated IR spectroscopy and MALDI-TOF MS analytical platform configures a robust, reliable system, cost-effective in terms of consumables and reagents, and fast in delivering the diagnosis. The spectral data of IR spectroscopy and MALDI-TOF MS obtained from these recovered patients can be evaluated with chemometrics’ tools, serving to build up an integrated spectral database. These data, after being validated with spectral molecular data of SARS-CoV-2 reference and clinical strains, can be used as a standard for the rapid detection of other individuals recovered from COVID-19.

This step is of utmost importance since it is necessary to quickly determine the individual groups that have already been exposed and that have recovered from COVID-19. In order to get individuals back into their social activities and to promote their mental health, as well as to boost economics, this platform appears as a cost-effective approach. In addition, as a strategy to known possible changes in the behavior of the virus and in the manifestation of the disease, the IR spectroscopy and MALDI-TOF MS spectral analysis may provide clues for emerging HCoVs and be an important contribution to the whole society.

The establishment of such a platform could allow a more efficient approach to control COVID-19 and also arboviruses infections collaborating to (1) diagnose, even if the viral load is low but sufficient to generate IR spectroscopy fingerprints and/or MALDI-TOF mass spectra biomarkers, at concentrations as low as 700 ppb and 10−15–10−18 mol/L, respectively; (2) quantify the viral load by IR spectroscopy of patients infected, and (3) diagnose recovered patients, using IR spectroscopy and MALDI-TOF mass spectrometry.

## Data Availability

Not applicable.
